# Progression of Subclinical Pachychoroid Neovasculopathy to an Active Neovascularization in the Presence of Acquired Vitelliform Lesions

**DOI:** 10.1155/2021/3098420

**Published:** 2021-11-09

**Authors:** Manoj Soman, Sameer Iqbal, Jay U. Sheth, Padmanaban Meleth, Unnikrishnan Nair

**Affiliations:** ^1^Vitreoretinal Services, Chaithanya Eye Hospital and Research Institute, Trivandrum, India; ^2^Chaithanya Innovation in Technology and Eyecare (Research), Trivandrum, India

## Abstract

We describe a unique case of bilateral acquired vitelliform lesions in a 67-year-old-female with pachychoroid associated with subretinal fluid in the right eye (OD) and a nonexudative choroidal neovascular membrane (CNVM) in the left eye (OS). Multimodal imaging performed at baseline and over the ensuing two years showed an increase in the OS vitelliform lesions with a concurrent transformation of quiescent CNVM to an exudative form. Further studies are warranted to gain better insight into the etiopathogenesis of these vitelliform lesions in pachychoroid and their potential role in instigating CNVM activation.

## 1. Introduction

Acquired vitelliform lesions (AVL) are a focal or multifocal subretinal accumulation of autofluorescent material reported in various dystrophic, degenerative, paraneoplastic, toxic, and vitreoretinal interface disorders involving the macula [1]. The natural course of these lesions is often a gradual decrease in size with fragmentation and slow resorption, resulting in photoreceptor disruption and eventual atrophy [2]. In this case report, we demonstrate that these acquired vitelliform deposits herald the transformation of a nonexudative choroidal neovascular membrane (CNVM) to an exudative one in a patient with pachychoroid.

## 2. Case Report

**Figure 1:**
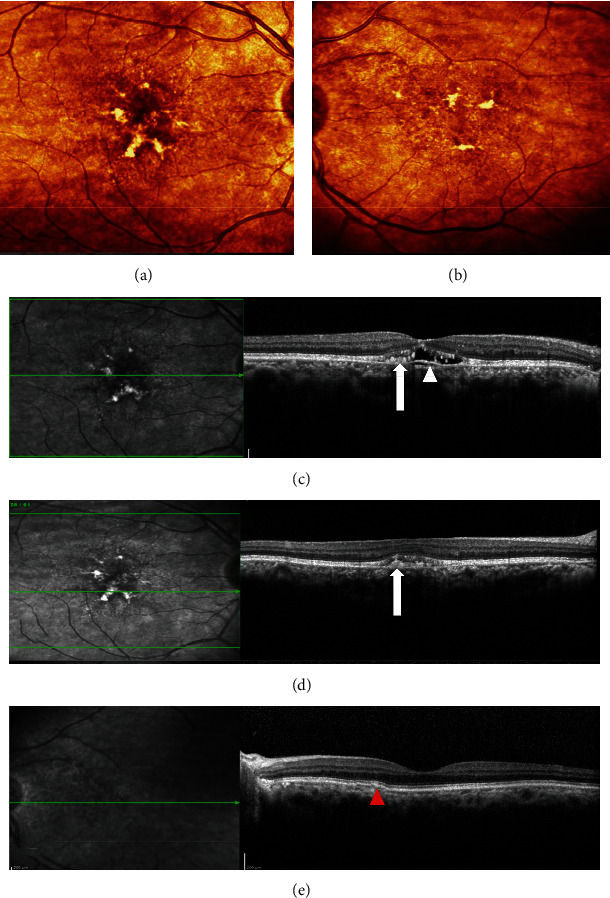
(a) and (b) Multicolour images (a) showing retinal pigment epithelial (RPE) alterations with vitelliform deposits in both macula simulating pattern dystrophy. Spectral-domain optical coherence tomography (SD-OCT) showing hyperreflective lesions in subretinal space ((c), (d)—arrows) with a shallow PED in the subfoveal region of the right eye ((c)—arrowhead) and similar deposition in the left eye (e) and focal extrafoveal DLS ((e) red arrowhead). Note the pachychoroid features in both eyes.

**Figure 2:**
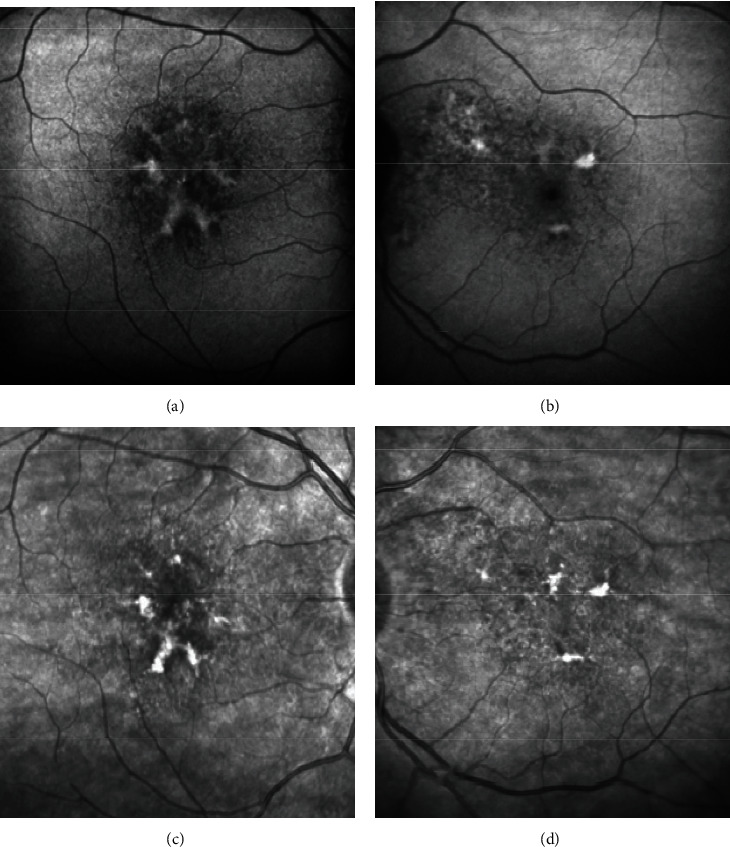
Blue peak autofluorescence imaging revealed increased autofluorescence from vitelliform deposits in both eyes ((a) and (b)) while infrared images showed corresponding increased reflectance ((c) and (d)).

**Figure 3:**
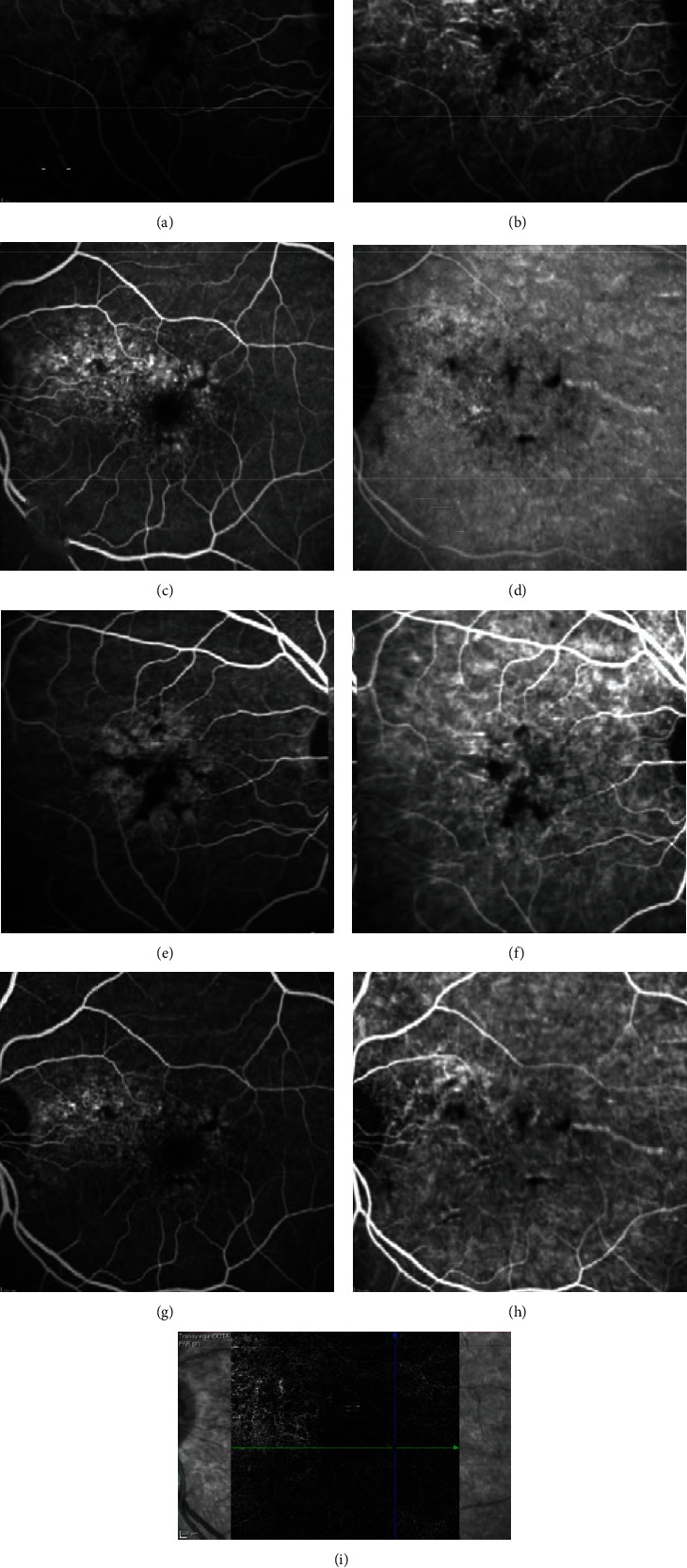
(a)–(h) Early and late phase combined fundus fluorescein angiography (FFA) and indocyanine green angiography (ICGA) showing window defects and blocked fluorescence corresponding to vitelliform deposits with no evidence of neovascularization network. Note the abnormal network evident on OCTA (i) in the left eye suggestive of nonexudative choroidal neovascular membrane (CNVM).

A 67-year-old lady with a history of hypertension, dyslipidemia, and ischemic heart disease presented to us with complaints of diminution of vision in the right eye for 1.5 years. Her best-corrected visual acuity (BCVA) was 20/40 in the right eye (OD) and 20/20 in the left eye (OS). OU anterior segment was normal. Fundus examination showed patterned retinal pigment epithelial (RPE) alterations in OD and nonspecific RPE changes in OS, seen better on multicolor imaging (Figures 1(a) and 1(b)). Vitelliform-like deposits were also seen in OU (Figures 1(a) and 1(b)). Spectral-domain optical coherence tomography (SD-OCT) revealed pachychoroid features with hyperreflective lesions in subretinal space and a shallow pigment epithelial detachment (PED) in OD (Figures 1(c) and 1(d)) and similar hyperreflective material extending into outer retinal layers and a focal extra-foveal double-layer sign (DLS; Figure 1(e)) in OS. Blue peak autofluorescence (BAF) imaging revealed increased autofluorescence from vitelliform deposits in both eyes (Figures 2(a) and 2(b)), while infrared images showed corresponding increased reflectance (Figures 2(c) and 2(d)). Combined fundus fluorescein angiography (FFA) and indocyanine green angiography (ICGA) showed window defects with foci of blocked fluorescence corresponding to the vitelliform lesions without any evidence of neovascularization (Figures 3(a)–3(h)). OCT angiography (OCTA) however revealed a nonexudative CNVM in OS corresponding to the focal DLS (Figure 3(i)). The patient was managed conservatively with scheduled follow-up visits.

**Figure 4:**
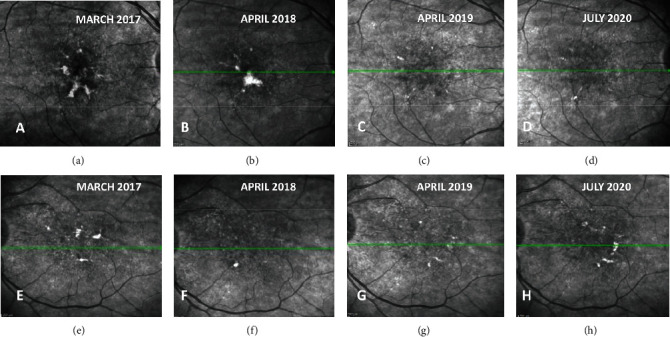
Serial infrared reflectance (IR) images demonstrating in the right eye (first row) central migration and gradual disappearance of vitelliform lesions and the left eye (bottom row) even though lesions disappeared initially, it reappeared at different locations in the macula.

**Figure 5:**
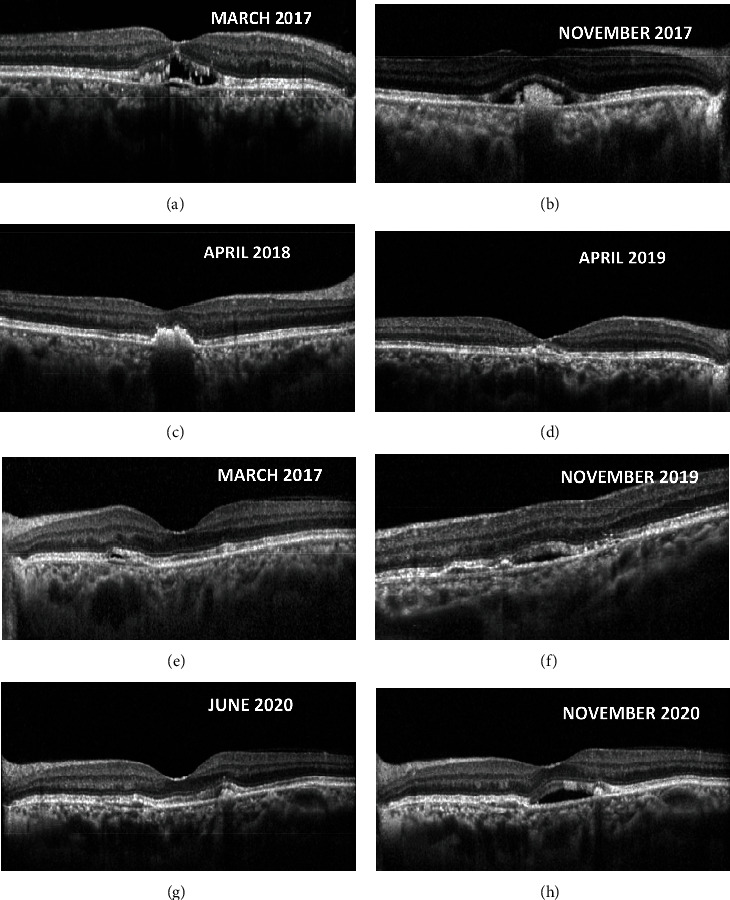
Serial spectral-domain optical coherence tomography (SD-OCT) images of right eye revealing flattening of pigment epithelial detachment (PED) (a) and central clumping of vitelliform deposits (b) and later resorption ((c) and (d)) while the left eye revealed focal double-layer sign (DLS; (e)) and development of extrafoveal fluid (f) managed conservatively and later new vitelliform deposition (g) followed by submacular fluid (h) necessitating treatment. Note that these new hyperreflective lesions appear before the onset of exudation (g).

**Figure 6:**
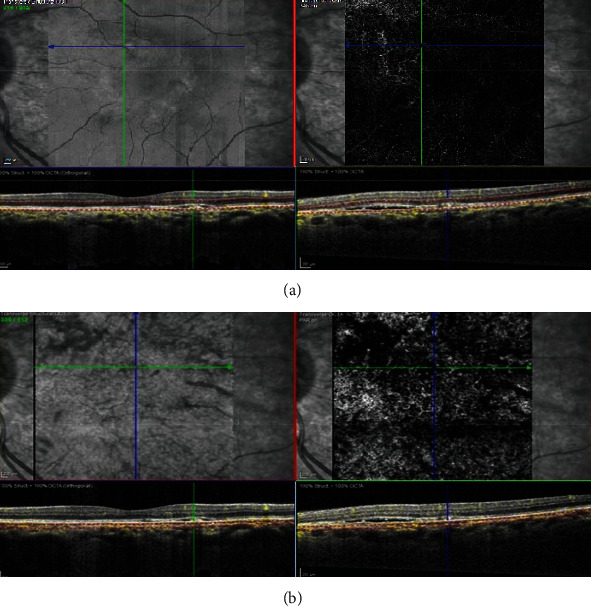
Optical coherence tomography angiography (OCTA) images of left eye corresponding to hyperreflective spot on infrared reflectance (IR) image showing an abnormal network in avascular layer and presence of prominent underlying and adjoining large dilated choroidal vascular complex.

**Figure 7:**
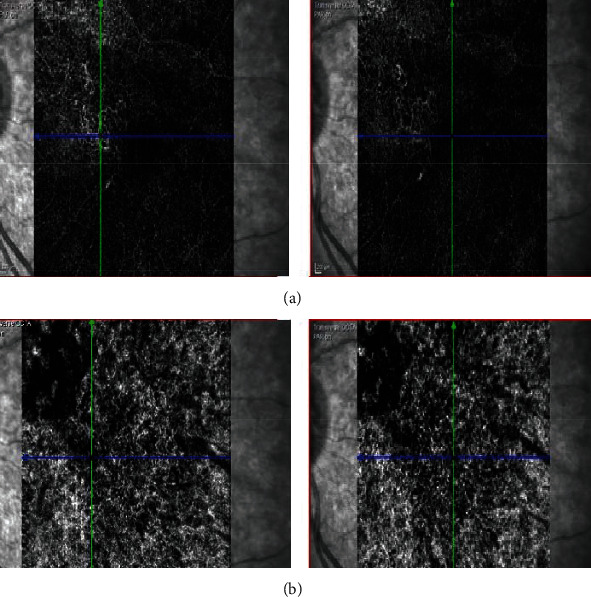
Comparison of baseline optical coherence tomography angiography (OCTA; nonexudative state) and OCTA at the time of submacular fluid (exudative state). Note the slight increase in the size of the vascular network in avascular slabs and the appearance of new dilated vessels in choroidal slabs.

**Figure 8:**
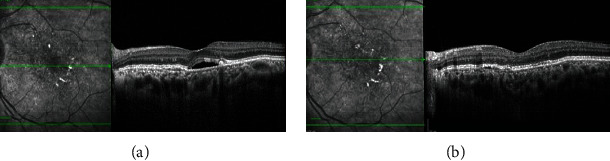
Pre- and posttreatment spectral-domain optical coherence tomography (SD-OCT) images ((a) and (b)) showing resolution of subretinal fluid and persistent reflective deposits on infrared reflectance (IR) imaging.

Serial infrared reflectance (IR; Figure 4) and SD-OCT (Figure 5) imaging over the next two years showed a gradual flattening of the PED with central migration of the vitelliform deposits and absorption in OD. The vision was maintained at 20/40 at all the visits in OD. In OS, the vitelliform deposits initially decreased only to reappear at different locations (Figure 4) with the onset of subretinal fluid (SRF; Figure 5). On colocalizing the vitelliform deposits with OCTA, we noted an abnormal network in the avascular slabs with dilated vessel complex in choroidal slabs suggestive of pachychoroid neovasculopathy (PNV; Figure 6). The size of the network had increased marginally as compared to baseline (Figure 7). Since the patient was symptomatic in OS with a decrease in BCVA to 20/40, she received two monthly doses of intravitreal ranibizumab injection and subsequently had complete resolution of SRF with BCVA improving to 20/20 (Figure 8).

## 3. Discussion

The purpose of this case report was to highlight the conversion of quiescent PNV to an exudative network with progressive deposition of AVL. We also illustrate the role of OCTA in detecting quiescent networks that are likely to be missed on dye-based angiography and for their periodic noninvasive monitoring. Additionally, intravitreal ranibizumab monotherapy can be successfully used to treat PNV in the presence of AVL.

In the current case, there was presence of bilateral pachychoroidopathy with AVL deposition. One eye showed spontaneous resolution of the deposits while the other eye having PNV demonstrated recurrent deposition with the onset of exudation. Freund et al. had reported features of AVL eyes demonstrating the natural history and imaging characteristics of these lesions [1]. 2.2% of the eyes in their series had CSCR and associated vitelliform lesions. Although these eyes could have associated pachychoroid features, this feature was not highlighted in the series. The presence of chronic SRF leads to loss of apposition between the photoreceptor tips and the RPE which can interfere with the phagocytosis of shed outer segments resulting in vitelliform deposition in these eyes [1, 3, 4]. In our case, the deposition seemed to appear along with or before the occurrence of SRF, especially in OS and was additionally associated with a neovascular network. Agawa et al. [5] recently reported findings in eyes with PNV but did not comment on such observations in the AF/IR imaging.

We believe that in pachychoroid eyes, the dilated outer choroidal vessels with a resultant choriocapillaris compression led to a primary RPE dysfunction. This can alter the physiological homeostasis of the RPE and neurosensory retina causing poor phagocytosis of the shed outer segments that can accumulate over time as the vitelliform lesions. Spontaneous resolution may occur when there has been sufficient photoreceptor loss to allow for the normal mechanisms of photoreceptor outer segment turnover to “catch up,” whereby RPE phagocytosis of the abnormal subretinal material may occur [1]. This phenomenon was demonstrable in the right eye of our case. However, the prevalent underlying choroidal neovascularization with episodes of activity in the left eye could have contributed to recurrent accumulation as seen in the left eye. Thus, unlike other causes of AVL, pachychoroid eyes could have recurrent vitelliform deposits. Also, the presence of vitelliform lesions reflective on near-infrared reflectance (nIR) reflectance suggests a high content of melanin, supporting a possible origin from the RPE monolayer in the form of RPE hyperplasia or macrophages containing large amounts of melanolipofuscin granules [6, 7]. Freund et al. [1] imaged some AVL eyes simultaneously with SD-OCT, autofluorescence, and nIR imaging and demonstrated near-infrared hyperreflectivity corresponding to presumed collections of vitelliform deposits, an observation also seen in our case.

Although many eyes having primary pattern dystrophy with vitelliform deposits are misdiagnosed as age-related macular degeneration (AMD), central serous chorioretinopathy (CSCR), or nonspecific RPE changes [8], the converse may also be a possibility. Entities such as pachychoroidopathy could present findings simulating pattern dystrophy [9]. The older age of the patient, absence of family history, asymmetrical features [10], appearances of recurrent new deposition, typical choroidal OCT features, and central migration of the deposits as seen in our case could differentiate this from primary pattern dystrophy.

In conclusion, our case highlights the presence of vitelliform lesions in pachychoroid eyes. We noted the transformation of a subclinical PNV to an exudative one with progressive deposition of vitelliform lesions. To the best of our knowledge, this is the first case report to describe the simultaneous occurrence of these two pathologies, namely, PNV and AVL. More research is needed to ascertain whether the simultaneous occurrence of these two pathologies is linked to each other or is a mere coincidence.
